# The role of different acetabular morphologies on patient-reported outcomes following periacetabular osteotomy in borderline hip dysplasia

**DOI:** 10.1007/s00402-024-05432-0

**Published:** 2024-07-05

**Authors:** Maximilian Fischer, Lars Nonnenmacher, Alexander Zimmerer, Johannes C. Reichert, Alexander Möller, Andre Hofer, Georg Matziolis, Georgi I. Wassilew

**Affiliations:** 1 Center for Orthopaedics, Trauma Surgery and Rehabilitation Medicine, University Medicine Greifswald, Greifswald, Germany; 2 Diakonieklinikum Stuttgart, Department of Orthopaedic and Trauma Surgery, Orthopädische Klinik Paulinenhilfe, Stuttgart, Germany; 3 Orthopaedic Department, Jena University Hospital, Campus Eisenberg, Eisenberg, Germany

**Keywords:** Borderline hip dysplasia, Developmental dysplasia of the hip, Periacetabular osteotomy, Hip preservation, Radiographic assessment, PROMs, Patient-reported outcome measure

## Abstract

**Introduction:**

The treatment option for borderline hip dysplasia (BHD) includes hip arthroscopy and periacetabular osteotomy (PAO). To the present day the controversial discussion remains, which intervention to prefer. Literature reports supporting an educated choice are scare, based on small patient cohorts and do not address the variability of acetabular morphology. Consequently, we intended to report PAO outcomes, from patients diagnosed with BHD, dependent on acetabular morphology, in a large patient cohort and aimed to define risk factors for poor clinical results and patient satisfaction.

**Materials and methods:**

A prospective monocentre study was conducted. Patients enrolled underwent PAO for symptomatic BHD (LCEA, 18°–25°). A total of 107 hips were included with 94 complete data sets were available for evaluation with a minimum follow-up of 1 year and a mean follow-up of 2.3 years. The mean age was 31 ± 8.2 years, and 81.3% were female. As the primary outcome measure, we utilized the modified Harris hip score (mHHS) with minimal clinically important change (MCID) of eight to define clinical failure. Results were compared after a comprehensive radiographic assessment distinguishing between lateral deficient vs. anterior/posterolateral deficient acetabular and stable vs. unstable hip joints.

**Results:**

Overall, clinical success was achieved in 91.5% of patients and the mHHS improved significantly (52 vs. 84.7, p < 0.001). Eight hips failed to achieve the MCID and four had radiographic signs of overcorrection. Comparing variable joint morphologies, the rate of clinical success was higher in patients with an anterior/posterolateral deficient acetabular covarage compared to lateral deficient acetabular (95.2% vs. 90.4%). tThe highest rate of clinical failure was recorded in unstable hip joints (85.7% vs. 92.5% in stable hips).

**Conclusions:**

This study demonstrates that PAO is an effective means to treat symptomatic BHD with variable acetabular morphologies, achieving a clinical success in 91.5% of all patients. To maintain a high level of safety and patient satisfaction technical accuracy appears crucial.

## Background

Borderline hip dysplasia (BHD) is a complex orthopaedic condition that involves subtle structural abnormalities of the hip joint. BDH has a prevalence of around 20% in the general population [1]. It is characterized by anatomical variations falling between normal acetabular coverage and classic hip dysplasia (HD). This acetabular undercoverage lead to increased biomechanical strain on the adjacent joint and soft tissue structures, culminating in progressive joint damage [2, 3].

Historically, developmental hip dysplasia was primarily defined using the Lateral centre-edge angle (LCEA), described by Wiberg [4]. In recent years, however, three-dimensional radiographic analyses have illuminated the various forms of acetabular coverage in hip dysplasia [5–7]. Additionally, radiographic parameters have been described to further evaluate not only the lateral acetabular coverage but also to determine acetabular undercoverage in anterior and posterior regions [8, 9]. Additionally, the femoro-epiphyseal-acetabular roof (FEAR) index was introduced by Wyatt et al. as a radiographic sign of joint instability in borderline hip dysplasia showing excellent intra- and interobserver reliability [10].

Despite these achievements, consensus remains elusive for the optimal treatment of patients with BHD [11]. Current therapeutic approaches focus on hip arthroscopy or bony correction through periacetabular osteotomy (PAO). Both procedures have demonstrated favourable results [11]. While some reports on hip arthroscopy revealed higher failure rates in BHD patients, the short-term follow-up of the more invasive PAO demonstrates safety and low revision rates [12–14]. However, it needs to be emphasized that available outcome reports of PAO in BHD are mainly based on small study cohorts [15, 16]. Solely, Nepple et al. reported the outcome after PAO for BHD in a larger patient cohort of 186 hips. The study results showed advantageous effects in patients undergoing primary PAO, while hips previously treated hip arthroscopically showed inferior outcomes [17]. Similarly, it is evident the postoperative results after hip arthroscopy of patients suffering from BHD are influenced by various hip morphology [18, 19]. Therefore, PAO could represent the preferable treatment modality for BHD independent of the individual acetabular configuration because PAO enables the correction of the osseous acetabular deformity using a minimally invasive approach in line with fast patient rehabilitation [20, 21]. Detailed analysis of PAO outcomes across variable types of acetabular and hip joint configurations in BDH are essential to further improve patient care.

Consequently, we set out to comprehensively assess pre- and postoperative radiographs in a cohort of > 100 patients treated for symptomatic BHD (LCEA 18°–25°) with emphasis on patient-reported outcomes contingent on different hip morphologies at a minimum follow-up of 1 year. We hypothesized that a precise bony deformity correction by PAO uniformly leads to improved postoperative patient-reported outcomes across various joint morphologies. Due to the fact that PAO enables the three-dimensional acetabular correction, it outlines a promising approach to address a wide spectrum of acetabular conditions with the aim to enhance patient-reported outcomes.

## Materials and methods

### Study design

We initiated a prospective follow-up study of 107 hips in 104 patients who underwent PAO due to symptomatic BHD (LCEA 18°–25°). The patients were treated at a single orthopaedic centre between 2019 and 2022.

**Fig. 1:**
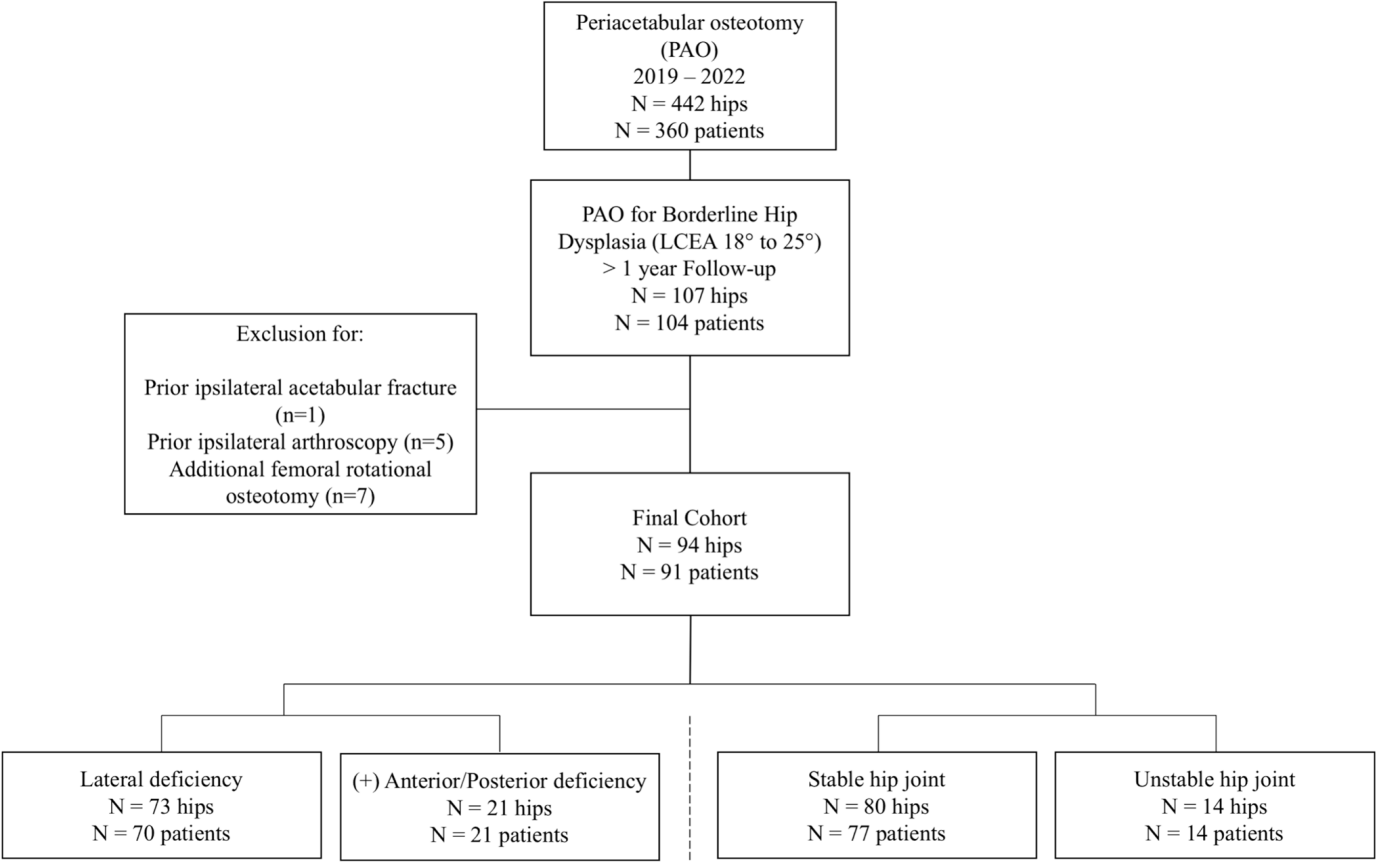
Hips that were included and excluded in this study and the subdivision into different hip joint morphologies

The patients presented with symptomatic and refractory hip pain lasting more than 6 months. BHD was diagnosed by a combination of patient reported symptoms, physical examination and radiographic parameters. Comprehensive radiographic diagnostic included X-rays of the anterior-posterior pelvis, as well as axial and oblique views of the affected hip joint. Indications for PAO were signs of osteoarthritis Tönnis grade < 2, a congruent hip joint, a history of therapy refractory hip pain in combination with radiographic signs of BHD. All patients gave written informed consent prior to study enrolment. Thirteen patients were excluded due to prior ipsilateral acetabular fracture (n = 1) and ipsilateral hip arthroscopy (n = 5) or the necessity of an additional femoral rotational osteotomy in the combination with the undergone PAO (n = 7) (Fig. 1).

### Surgical technique

A modified, minimally invasive Bernese periacetabular osteotomy (PAO) technique was performed in all patients. This involved the use of a bikini incision and either a rectus-sparing (RS) approach (46/94) with bony detachment of the sartorius from its origin or a rectus- and sartorius-sparing (RASS) approach (48/94) as previously described by our group [22]. A mini-open arthrotomy was performed for a femoral head-neck osteochondroplasty in case of femoral asphericity right after PAO using the Smith-Peterson approach. All procedures were performed by the senior author (GIW). Physiotherapeutic training and mobilization of the operated hip joint started directly on the first postoperative day. In case of RASS approach, active hip flexion was permitted directly after surgery.

Complications and reoperations were assessed by consecutive patient follow-up. Implant removal was performed in 31 patients (34%) within the follow-up period and was not graded as intervention due to surgical complications.

### Radiographic assessment

Radiographic assessment was performed by the first and senior authors. Preoperative and postoperative anterior-posterior pelvic X-rays were reviewed independently to analyse lateral centre-edge angle (LCEA). Further parameters included the, Tönnis osteoarthritis grade, medial centre-edge angle (MCEA), acetabular inclination (AI), anterior- and posterior wall index (AWI/PWI), signs of acetabular retroversion (crossover, posterior wall sign, sciatic spine sign), FEAR-index and Sourcil upsloping.

**Fig. 2:**
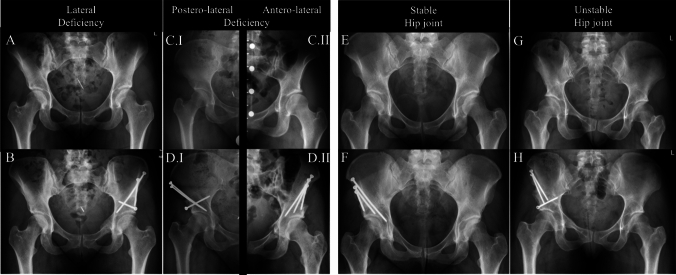
Representative patient cases with different acetabular and hip joint morphologies: pre-(A/C/E/G) and postoperative (B/D/F/H) anterior–posterior pelvic radiographs

According to the radiographic assessment hip morphology was allocated to four distinct clusters—I.I lateral deficiency (LCEA 18°–25°, normal AWI/PWI) or I.II anterior/posterior—lateral deficiency (LCEA 18°–25°, low AWI/PWI) – II.I stable hip joint (LCEA 18°–25°, FEAR-index < 2°) or II.II unstable hip joint (LCEA 18°–25°, FEAR-index > 2°) (Fig. 2).

### Data collection

Data was collected from electronic medical records, including patient demographics, preoperative comorbidities and operative details. The functional outcome after surgery was assessed by the modified Harris Hip Score (mHHS), International Hip Outcome Tool-12 (iHOT-12), University of California Los Angeles Activity scale (UCLA) and the Hip disability and Osteoarthritis Outcome Score (HOOS). In addition, the minimal clinically important difference (MCID) for the primary outcome measure, the mHHS, was analysed. A MCID of ≥ 8 was determined as a clinically meaningful change [23].

### Statistical analysis

Descriptive statistics were used to summarize the patient characteristics and outcomes. Radiographic and patient reported outcome data were reported as mean with standard deviation. Statistical analysis and dataset presentation were performed using GraphPad Prism 9.4.1. A two-tailed paired t-test was used to compare pre- and postoperative patient-reported outcome and a two-tailed unpaired t-test was used to compare postoperative results between the groups. A p-value less than 0.05 was considered statistically significant.

### Ethical considerations

All patients gave written informed consent prior to inclusion. Ethics approval (BB099/20) was obtained from the local independent ethics committee (IEC) of the University Medicine Greifswald according to the World Medical Association Declaration of Helsinki.

## Results

### Patient characteristics and preoperative radiographic parameters

A total of 107 hips in 104 patients were initially enrolled in this study. Thirteen patients did not fulfil the inclusion criteria due to prior ipsilateral acetabular fracture (n = 1), prior ipsilateral hip arthroscopy (n = 5) or the necessity of an additional femoral rotational osteotomy (n = 7) bringing the data set to a total of 94 hips in 91 patients. The mean age at the time of PAO was 31 ± 8.2 years, the mean BMI 24.7 ± 4.5. Seventeen patients (18.7%) were male, 74 (81.3%) were female. The mean follow-up was 2.3 ± 0.9 years.

The preoperative osteoarthritis assessment of the study cohort revealed 53 hips (56.4%) with Tönnis grade 0 and 41 hips (43.6%) with Tönnis grade 1. At the time of the surgical procedure, the mean LCEA was 20.4° ± 2.4° and the mean AI was 8.2° ± 4.4°. Acetabular retroversion was observed in 28.7% (27/94) of the included hips, based on the presence of all radiographic signs characteristics regarding acetabular retroversion (crossover sign, posterior wall sign, sciatic Spine sign) (Table 1).

**Table 1:** Patient characteristic and preoperative radiographic parameters

	Total	Lat. deficiency	(+) Ant/post. deficiency	Stable	Unstable
n (hips) n (patients)	94 91	73 70	21 21	80 77	14 14
Age ± SD	31 (± 8.2)	31.4 (± 8.1)	29.5 (± 8.3)	31.3 (± 8.2)	29.4 (± 8.3)
% female	81.3	81.4	81	79.2	92.8
Mean BMI ± SD	24.7 (± 4.5)	25.1 (± 4.6)	23.4 (± 4)	25.1 (± 4.6)	22.3 (± 3.1)
Median Tönnis grade (min–max)	0 (0–2)	0 (0–1)	0 (0–2)	0 (0–2)	0 (0–1)
LCEA (° ± SD)	20.4 (± 2.3)	20.5 (± 2.3)	20 (± 2.1)	20.7 (± 2.5)	18.7 (± 0.7)
MCEA (° ± SD)	35.3 (± 5.7)	35 (± 5.9)	36.1 (± 5.1)	35 (± 5.4)	36.9 (± 4.4)
Acetabular Inclination (° ± SD)	8.2 (± 4.4)	7.8 (± 4.4)	9.4 (± 4.2)	7.7 (± 3.5)	10.8 (± 3.1)
Acetabular retroversion signs n					
Crossover	44	35	9	36	8
Posterior wall sign	56	45	11	46	10
Sciatic spine	28	22	6	24	4
All three signs	27	21	6	23	4
AWI (% ± SD)	42.7 (± 10.3)	45.6 (± 7.6)	32.8 (± 12.3)	42.7 (± 7.4)	42.7 (± 14.7)
PWI (% ± SD)	88 (± 15.5)	89.1 (± 13.2)	84.1 (± 21.2)	88.6 (± 9.2)	84.2 (± 12.7)
FEAR (° ± SD)	− 5.9 (± 8.1)	− 5.4 (± 7.8)	− 7.5 (± 8.9)	− 8 (± 5.7)	6.4 (± 4.1)
Sourcil upsloping n (%)	48 (51)	40 (54.8)	8 (38.1)	42 (52.5)	6 (42.8)

*Cluster I.I—lateral deficiency* included 73 hips (77.7%) in 70 patients. The preoperative mean LCEA in this sub-group was 20.5 ± 2.3° with a mean AI of 7.8 ± 4.4°. The preoperative mean AWI was 45.6 ± 7.6% and the mean PWI equaled 89.1 ± 13.2% (Table 1).

*Cluster I.II—anterior/posterior-lateral deficiency* consisted of 21 hips (22.3%) in 21 patients. 14 hips exhibited a anterolateral deficient femoral head coverage and 7 hips showed a posterolateral acetabular deficiency. The preoperative mean LCEA in this subset was 20.0 ± 2.1° with a mean AI of 9.4 ± 4.2°. The preoperative mean AWI was 32.8 ± 12.3% and the mean PWI 84.1 ± 21.2%. In 28.6% (6/21 hips) all signs of acetabular retroversion were present (Table 1).

*Cluster II.I—stable hip joint* embraced 80 hips (85.1%) in 77 patients. The preoperative mean LCEA in this group measured 20.7 ± 2.5° with a mean AI of 7.7 ± 3.5°. The preoperative mean FEAR-index was –8.0 ± 5.7° (Table 1).

*Cluster II.II—unstable hip joint showed* 14 hips (14.9%) in 14 patients. The preoperative mean LCEA in this group was 18.7 ± 0.7° with a mean AI being 10.8 ± 3.1°. The preoperative mean FEAR-index was 6.4 ± 4.1° (Table 1).

### Surgical procedure and postoperative radiographic parameters

In 51.1% (48/94 hips) a RS approach was performed and a RASS approach was used in 48.9% (46/94 hips) of all cases. A femoral head-neck osteochondroplasty in case of femoral asphericity was additionally performed in 79.8% (75/94 hips) (Table 2). The median number of screws used for osseous fixation was three.

**Table 2:** Surgical technique and postoperative radiographic parameters

	Total	Lat. deficiency	(+) Ant/Post. deficiency	Stable	Unstable
RASS approach n (%)	46 (48.9)	35 (47.9)	11 (52.4)	38 (47.5)	8 (57)
Median screws n (min–max)	3 (2–4)	3 (2–4)	3 (3–4)	3 (2–4)	3 (2–4)
Concurrent arthroscopy n (%)	11 (11.7)	9 (12.3)	2 (9.5)	9 (11.2)	2 (14.2)
LCEA (° ± SD)	30.2 (± 3.9)	30.4 (± 4)	29.6 (± 3.7)	30.4 (± 3.6)	29.1 (± 2.6)
MCEA (° ± SD)	27.7 (± 5.8)	27.7 (± 6.1)	27.9 (± 4.6)	27.9 (± 5.3)	26.7 (± 6.6)
Acetabular Inclination (° ± SD)	0.1 (± 4)	0.1 (± 4.2)	0.4 (± 3.1)	0 (± 4.3)	0.8 (± 4.3)
AWI (% ± SD)	40 (± 11.1)	41.4 (± 10.5)	35.1 (± 11.5)	39.5 (± 13.9)	42.3 (± 10.3)
PWI (% ± SD)	96.8 (± 13.7)	97.3 (± 11.9)	95.2 (± 18.6)	98.3 (± 10.8)	88.5 (± 13.4)
FEAR (° ± SD)	− 14.6 (± 6.6)	− 14.5 (± 6.8)	− 14.9 (± 6.1)	− 16.1 (± 5.9)	− 5.9 (± 6.3)

Considering the entire study cohort, the mean LCEA has improved to 30.2° ± 3.9° and the mean AI hat improved to 0.1° ± 4° following the surgical procedure. No significant difference was observed between the defined subgroups (Table 2).

In patients with preoperative radiographic anterolateral/ posterolateral deficiency (cluster I.II), the AWI and PWI has improved postoperatively. Evaluating cluster II, an improvement in the FEAR Index, due to surgical correction, was observed in unstable hip joints (Table 2).

### Patient-reported outcomes

**Fig. 3:**
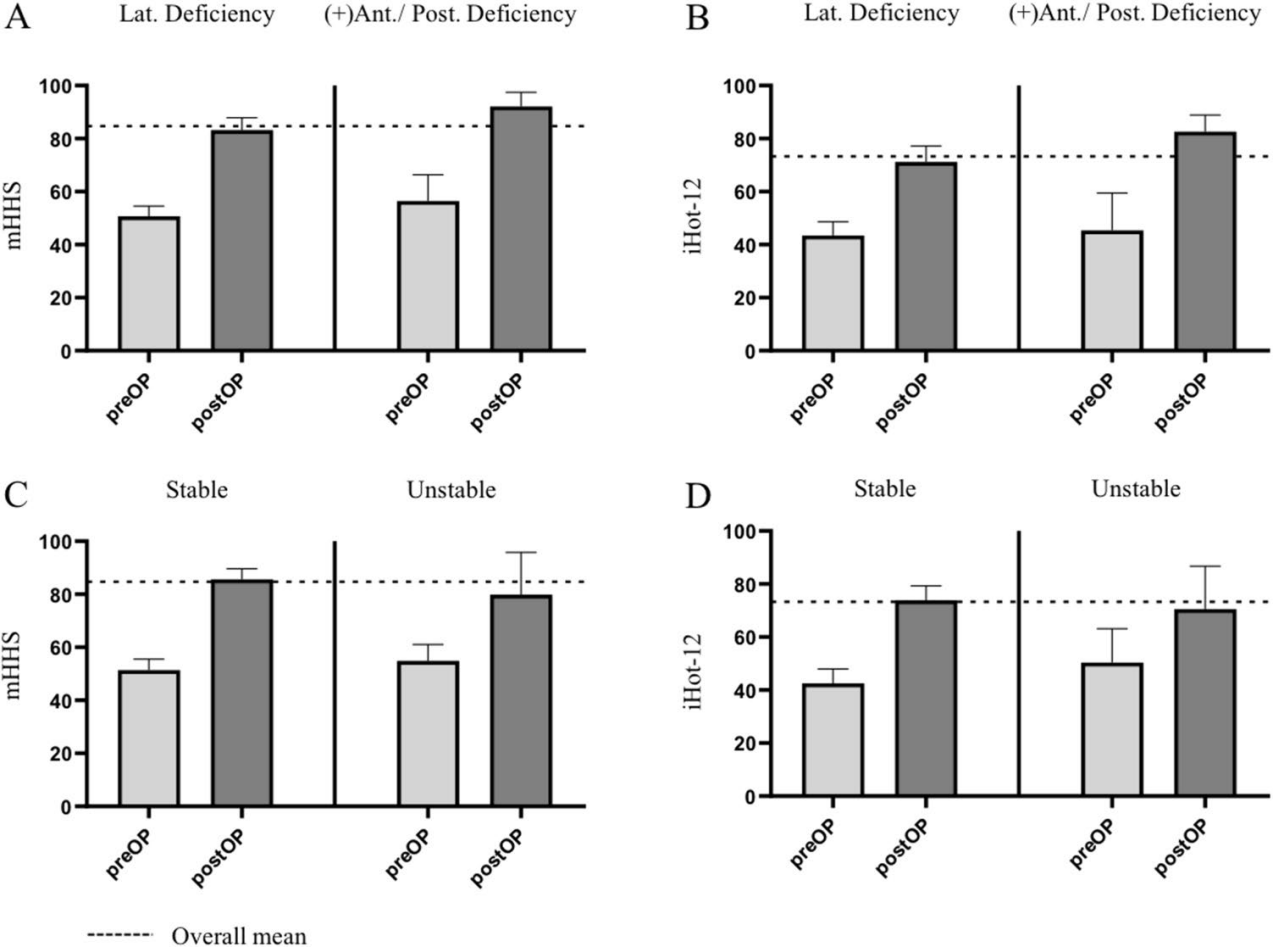
**A**/**C** Modified Harris hip score (mHHS—**A**/**C**) and International Hip Outcome Tool-12 (iHOT-12—**B**/**D**) for different acetabular morphologies, – Mean ± standard deviation

At the latest follow-up, the patient-reported outcome measures had significantly improved across the study cohort compared to preoperative values. These findings include the mHHS and the iHot-12 scores (Fig. 3) as well as the UCLA and the HOOS domains – pain, sports, symptoms, activity in daily living and quality of life. Overall, 91.5% of patients achieved the mHHS MCID after the surgical procedure (Table 3).

**Table 3:** Patient-reported outcome measures—mean ± standard deviation, *statistically significant, p < 0.05

	Total	Lat. deficiency	(+) Ant/post. deficiency	Stable	Unstable
mHHS					
Preoperative	52 (± 17)	50.7 (± 15.8)	56.4 (± 20.1)	51.4 (± 17.9)	54.9 (± 10.3)
Latest follow-up	*84.7 (*± 17.5)*	*83.2 (*± 18.5)*	*92 (*± 8.3)*	*85.6 (*± 15.8)*	*79.8 (*± 24)*
MCID (8)	91.5%	90.4%	95,2%	92.5%	85.7%
iHOT-12					
Preoperative	43.8 (± 20.1)	43.3 (± 19.1)	45.4 (± 23.5)	42.5 (± 20.2)	50.4 (± 18)
Latest follow-up	*73.3 (*± 19.6)*	*71.2 (*± 20.7)*	*82.8 (*± 8.8)*	*73.9 (*± 18.8)*	*70.7 (*± *25.3) n.s*
UCLA					
Preoperative	6.2 (± 2.5)	6.3 (± 2.5)	6.0 (± 2.2)	6.1 (± 2.5)	6.8 (± 2.3)
Latest follow-up	*6.9 (*± 2.0) *	*6.7 (*± *2.1) n.s*	*7.7 (*± 1.1) *	*6.9 (*± 1.9) *	*7 (*± *2.4) n.s*
HOOS					
Pain					
Preoperative Latest follow-up	48.7 (± 21) *78.9 (*± *17.8*) *	48 (± 19.9) 78.1 (± 18.9)*	51.5 (± 24.7) 83.7 (± 10.1)*	47(± 21) 78.4 (± 18.5)*	57.9 (± 18.6) 81.6 (± 13)*
Sport					
Preoperative	40.7 (± 26.2)	39.3 (± 24.4)	46.1 (± 31.6)	40 (± 25.7)	47.9 (± 26.6)
Latest follow-up	*72.3 (*± *21.7*) *	69.3 (± 24.1) *	81.9 (± 12.2)*	72.2 (± 21.8)*	72.5 (± 21.1)*
Symptom					
Preoperative	54.5 (± 22.5)	54.2 (± 22.3)	55.9 (± 23.4)	52.5 (± 22.8)	65.8 (± 17.3)
Latest follow-up	*73.3 (*± *17.7*) *	72.5 (± 17,9)*	78.2 (± 15.6)*	73.5 (± 17.8)*	72.3 (± 17.5) n.s
Activity of daily living					
Preoperative	62.4 (± 24)	61.3 (± 23)	66.7 (± 27.2)	61.2 (± 25.3)	73.5 (± 18.9)
Latest follow-up	*84.9 (*± *14.9*) *	83.6 (± 15.6)*	91.6 (± 7.7)*	85.5 (± 13.9)*	81.3 (± 19) n.s
Quality of life					
Preoperative	29.5 (± 18.9)	28.7 (± 19.2)	31.3 (± 17.7)	28.8 (± 18.9)	33.3 (± 17.7)
Latest follow-up	*58.7 (*± *25.3*) *	56.5 (± 26.7)*	65.9 (± 19.1)*	59.8 (± 24.5)*	52.5 (± 27.6)*

In the subgroup analyses these findings were consistent in patients with radiographic signs of isolated lateral deficiency as well as in patients with radiographic signs of combined anterolateral/ posterolateral deficiency. The rate of patients achieving the mHHS MCID was higher in patients with combined anterolateral/posterolateral deficiency compared to isolated lateral deficiency (95.2% vs. 90.4%) postoperatively (Table 3).

Considering stable and unstable hip joints, the patient-reported outcome measures had significantly improved for stable hip joints. Patients with unstable hip joints showed a significant postoperative improvement for the mHHS and the HOOS scores regarding pain, sports and quality of life. Additionally, patients with stable hip joints achieved the mHHS MCID (92.5% vs. 85.7%) more often when compared to patients with unstable hip joints (Table 3).

### Clinical failure analyses

Overall, eight hips in eight patients failed to achieve the mHHS MCID at the latest follow-up and were defined as clinical failure. The patients demographics did not differ from those of the remaining study cohort. The most commonly reported symptom was persisting pain (5/8) at the latest follow-up and two patients underwent a subsequent hip operation due to a unsatisfying outcome after the initial PAO. Half (4/8) of the patients with clinical failure had a LCEA > 30° combined with an AI of < 0° in the postoperative radiographic assessment (Table 4).

**Table 4:** Overview of patients failing to achieve the mHHS minimal clinical important difference (8)

n (hips) n (patients)	8 8
Age ± SD	30.7 (± 9)
% female	62.5
Mean BMI ± SD	23.9 (± 3.6)
Persisting pain n (%)	5 (62.5)
Subsequent operation n (%)	2 (25)
Postoperative LCEA > 30° n (%)	4 (50)
Postoperative AI < 0° n (%)	5 (62.5)
Postoperative LCEA > 30° and AI < 0° n (%)	4 (50)

## Discussion

This study illustrates that PAO significantly improved clinical symptoms and outcome scores across a variety of acetabular and joint morphologies with high patient safety in BHD. While the results demonstrate superior clinical outcomes in patients with anterolateral/posterolateral acetabular deficiency as well as for isolated lateral acetabular deficiency, the comparison of radiographically stable and unstable hip joints showed inferior postoperative outcomes for unstable hip joints.

To date, the optimal surgical therapy for patients in BHD remains a topic of debate. Studies on arthroscopy, PAO or their combination have reported improvement in clinical symptoms. While studies on radiographic and morphologic risk factors for poor outcome after arthroscopy are available, a comprehensive evaluation of clinical outcomes after PAO dependent on acetabular and hip joint morphologies is still lacking [18, 19, 24].

The most commonly used radiographic parameter to assess femoral head coverage is the LCEA of Wiberg [4]. In recent years, several additional radiographic parameters have been explored to characterize the acetabular and hip joint morphology more precisely [25–27]. While McClincy et al. identified gender specific acetabular subtypes in BHD, this study focused on different radiographically determined joint morphologies without gender differentiation [26]. Dornacher et al. reported up to 40% of hips with BDH combined with an anterior or posterior acetabular deficiency in their patient cohort [28], while the results of this study showing a proportion of 22.3% hips with substantial anterolateral or posterolateral acetabular deficiency. Thus, a thorough preoperative radiographic assessment is essential to facilitate a precise analysis of acetabular morphology.

Hip dysplasia and BDH have primarily been treated through acetabular reorientation via PAO or hip arthroscopy targeting intraarticular pathologies and CAM deformity [13, 29, 30]. In hip arthroscopy varying degrees of failure accompanied by a relatively high rate of reoperation have been reported [31, 32]. In this context, studies have highlighted limitations of hip arthroscopy, where a higher acetabular inclination, broken Shenton line, or acetabular retroversion correlated with higher revision rate or poor patient-reported outcomes [18, 19, 24]. In contrast, studies on PAO-treated borderline hip patients reported improved patient-reported outcomes and low complication and revision rates postoperatively [15, 17]. Recently, a small retrospective study in 42 hips with BHD reported improved patient-reported mid- to long-term outcomes of PAO for the first time [33]. In addition, the present study showed improved outcome after PAO in BHD with a minimum follow-up of 1 year.

When comparing the various acetabular and joint morphologies radiographically, unstable hip joints less frequently achieved the mHHS MCID (85.7% vs. 92.5% in stable hip joints) postoperatively. The FEAR-index, with a cut-off value of 2°, predicts hip instability with 90% probability on plain radiographs [10, 34]. Besides osseous undercoverage in anterior, lateral or posterior acetabular regions, BHD often includes labral and chondral comorbidities [35]. Since hip stability primarily depends on the osseous geometry, an osseous correction by PAO is recommended in case of instability. Additionally, studies have shown that distinct arthroscopic procedures may further worsen joint instability [36, 37]. While no correlation between the preoperative FEAR- index and the patient-reported outcome after PAO has been demonstrated [33], further research should investigate this aspect to enhance therapeutic approaches and surgical recommendations.

Despite the high overall success rate of over 90% reported in this study, eight patients failed to achieve the mHHS MCID and were defined as a clinical failure. Half of these patients exhibited postoperative radiographic signs of overcorrection (postoperative LCEA > 30° combined with a negative AI), supporting findings of Andronic et al. that reported an overcorrection in 66% of clinically failed cases and its negative correlation with patient-reported outcomes [33]. Thus, overcorrection could be a risk factor for poor outcome following PAO in BHD and must be evaluated in further studies.

While the present study reports improved patient-reported outcome in different joint subtypes, several limitations of this study must be considered. The study included only patients receiving PAO to treat BDH and there could be an increased risk for selection and treatment bias caused by a single surgeon performing all treatments in this study. A control group of patients receiving hip arthroscopy, or both was not available yet. Thus, a prospective cohort study is planned to compare hip arthroscopy and PAO in patients with BHD. Additionally, multi-centre studies could be beneficial to improve the generalizability of the results. Next, the data were limited by a minimum follow-up of 1 year. Thus, mid- and long-term outcomes have to be reported in future studies. Although a comprehensive radiographic assessment to describe the femoral and acetabular morphology was performed, most of the radiographic parameters were measured on plain pelvic X-rays, while MRI and femoral torsion measurements were not available in all patients of the study cohort. Therefore, results on accompanying soft tissue pathologies of the hip joint and femoral torsion affecting the outcome after PAO must be stated preliminary and must be further evaluated. Last, the group of patients with anterior/posterior-lateral deficiency included only 21 hips (14/7 hips), limiting a representative outcome assessment. Thus, future studies on this subgroup are planned to improve the clinical evidence in this distinct acetabular morphology.

Overall, the present study demonstrates significant clinical improvements across variable acetabular morphologies after periacetabular osteotomy in Borderline hip dysplasia. Appropriate experience in periacetabular osteotomy in combination with technical accuracy is crucial to maintain the high level of patients outcomes and safety. Further research is warranted to improve treatment recommendations, particularly regarding unstable hip joint conditions in Borderline hip dysplasia.
